# Assessment of country policies affecting reproductive health for adolescents in the Philippines

**DOI:** 10.1186/s12978-018-0638-9

**Published:** 2018-12-12

**Authors:** Junice L. D. Melgar, Alfredo R. Melgar, Mario Philip R. Festin, Andrea J. Hoopes, Venkatraman Chandra-Mouli

**Affiliations:** 1 Likhaan Center for Women’s Health, Quezon City, Philippines; 2 Department of Reproductive Health and Research/ Special Programme of Research, Development and Research Training in Human Reproduction, World Health Organization, Geneva, Switzerland; 3 Adolescent Center, Kaiser Permanente Washington, Seattle, Washington USA

## Abstract

**Background:**

Adolescents in the Philippines face many legal, social and political barriers to access sexual and reproductive health (SRH) services, putting them at higher risk of unplanned pregnancy, abortion, sexually transmitted infections and HIV, and other health and development problems.

**Objective:**

This study aims to evaluate whether current normative documents on SRH in the Philippines are in concurrence with adolescents’ human rights principles using the World Health Organization (WHO) Guidance and Recommendations on ensuring human rights in the provision of contraceptive information and services.

**Methods:**

The review focused on policies and normative guidance documents which included the national reproductive health law, its implementing rules and regulations, and the Supreme Court decisions on the law, and documents cited in the government’s Adolescent and Youth Health Programme. Also included were documents identified through keyword searches in an online database of the health department. We assessed these documents on their agreement or non-agreement with WHO recommendations, and the presence or absence of adolescent-specific content.

**Results:**

Of nine WHO summary recommendations, Philippine normative documents are in agreement with four, namely on acceptability, participation, accountability, and quality, and have adolescent-specific provisions in three. Philippine normative documents are partly in agreement with the remaining five WHO summary recommendations—nondiscrimination, availability, accessibility, informed decision-making, and privacy. Of twenty-four WHO sub-recommendations, Philippine normative documents are in agreement with fifteen, not in agreement with five, and partly in agreement with four. Two possible factors may explain the many documents with conflicting contents: devolution of the Philippine health system, and the deep social and policy divide on sexual and reproductive health.

**Conclusion:**

Many Philippine-governmental norms and standards are in agreement with adolescents’ human rights to contraceptive information and services as recommended by the WHO. However, a significant number are restrictive, reflecting the strong influence of conservative religious beliefs.

**Recommendations:**

We recommend: 1) further elaboration of the laws and policies that are fully in agreement with WHO recommendations; 2) a more liberal interpretation of the law to ensure the provision, delivery and access to reproductive health care services, and to promote, protect and fulfill women’s reproductive health and rights; and 3) popularization of ethical and human rights norms.

## Plain English summary

Adolescents in the Philippines are prevented from full access to sexual and reproductive health (SRH) services by legal obstacles, social and cultural restrictions, and their lack of meaningful political power. This exposes them to SRH problems like unplanned pregnancy, abortion, sexually transmitted infections and HIV— all with serious health and social consequences.

This study aims to assess whether the current laws and policies on SRH in the Philippines respond to the needs and rights of adolescents to contraceptive information and services. It uses a WHO document providing guidance and recommendations on ensuring human rights in the provision of contraceptive information and services.

We reviewed a new law on reproductive health and its implementing rules, Supreme Court decisions on the law, documents of the national adolescent health programme, and other policies and guidance from the Department of Health. We assessed if Philippine laws and policies are in agreement with WHO recommendations, and if these are specific or relevant to adolescents. The study results show that Philippine laws and policies are in agreement with four of nine WHO summary recommendations—acceptability, participation, accountability, and quality; and partly in agreement with five—nondiscrimination, availability, accessibility, informed decision-making, and privacy. We also found that of the 24 WHO sub-recommendations, Philippine laws and policies are in agreement with 15, partly in agreement with 4, and not in agreement with 5. The latter norms reflect the strong influence of conservative beliefs that look at contraceptives as inherently wrong and improper for adolescents’ use.

The study urges the implementation of laws and policies in agreement with the WHO recommendations. It also urges a more liberal interpretation of the law to ensure SRH access and the protection and promotion of girls’ and women’s reproductive health rights. A final recommendation is to explore changing the law while popularizing ethical and human rights norms.

## Introduction

Adolescents in the Philippines, both unmarried and married, face many sexual and reproductive health risks stemming from early, unprotected, and/or unwanted sexual activity [1]. Adolescent girls are particularly vulnerable to unintended pregnancies and maternal morbidity and mortality, including sequelae arising from unsafe abortions. Young parents often have to stop their education, limiting employment opportunities as adults. Policies that ensure and improve adolescents’ access to contraceptive information and services can reduce these health and social problems. This article examines how the Philippines’ new reproductive health law, Supreme Court rulings, and related policies impact on the specific needs of adolescents.

Among women between age 15–19, 10.1% report having been pregnant in 2013, up from 6.5% in 1993. The annual birth rate in this age group has remained almost constant in the last 20 years— from 50 births per 1000 in 1993 to 57 in 2013. In sharp contrast, all other age groups recorded steady declines in the same period. [2]. The country’s teen birth rate is currently higher than the average of 40 per 1000 for the South East Asian region and 15 per 1000 for the Western Pacific region [3].

Most adolescents report practicing abstinence as their main method to avoid pregnancy. However, this behavior is changing slowly towards more engaging in sexual activity. Among teenage women 15–19, those reporting ever having sex rose from 9.1% in 1993 to 14.7 in 2013. Modern contraceptive use in this age group also rose from 0.7 to 2.4% in the same period. While contraceptive use may be increasing, the prevalence rate is still low compared to the proportion of adolescents already having sex. Low contraceptive use persists even among adolescents who are married formally or in informal unions. In 2013, among all age groups of married women, adolescents had the lowest rate of use at 20.6% and the highest unmet need at 28.7% [2].

Adolescent pregnancies contribute to maternal deaths [4, 5]. Although the methods used in the country [6, 7] cannot accurately measure maternal mortality by age groups, it is generally accepted that preventing unintended pregnancies can prevent maternal deaths [5]. Adolescents are particularly at risk because they have less access to contraceptive services [8]. Adolescent pregnancies have been shown to contribute to early childhood deaths. According to the 2013 demographic and health survey (DHS), “mother’s age less than 18 (risk ratio = 2.13) is the single factor most associated with increased risk of under-5 mortality in the Philippines.” [2].

The government started a population growth reduction programme in the late 1960s with the goal of promoting socio-economic development. Fertility reduction through modern contraception was the primary strategy [9]. This phase lasted until the mid-1980s almost entirely under a martial law government. After the regime was ousted in 1986, a period of policy drift followed [10]. The Catholic hierarchy played a crucial role in the ouster and became very influential in the new government that took over and began to limit government programmes on contraception and family planning. Around this time, the International Conference on Population and Development took place in 1994, introducing a new framework of reproductive health and rights and dropping the centrality of population growth reduction. The Department of Health (DOH) of the Philippines adopted this framework in 1998 and there were various attempts at resuming and strengthening a national population and reproductive health programme. In the ensuing years, however, conservatives repeatedly defeated efforts to pass a law on reproductive health and rights that would ensure public funding and sustainability of this work.

In December 2012, after years of campaigning by civil society organizations, the “Responsible Parenthood and Reproductive Health Act” or reproductive health (RH) law was finally enacted [11]. For adolescents, the RH law mandated the provision of comprehensive sexuality education. On contraceptive services, however, the law required parental consent for minors, unless the adolescent had been pregnant before. Three months later, the Supreme Court halted the implementation of the law after conservative groups challenged its constitutionality. It took more than a year for the Supreme Court to rule that the RH law is generally constitutional, except for eight provisions that it nullified. Among the provisions removed is the parental consent exception for minors with a previous pregnancy [12].

Given the evolution of the legislative context through the past few decades, this study aims to evaluate whether current Philippine policies agree with the human rights framework developed by WHO on access to quality contraceptive information and services. This first attempt to review and analyze policies on adolescents’ sexual and reproductive health in the Philippines would allow a deeper insight on how these various historical events and policy changes have affected the present national normative guidelines that would determine the range and quality of services available to the clients, with adolescents in particular.

## Methods

In 2014, WHO published “Ensuring Human Rights in the Provision of Contraceptive Information and Services” [13], which aims to provide guidance on priority actions that should be taken to ensure that the different human rights dimensions are systematically and clearly integrated into the provision of contraceptive information and services. It is based on evidence that the respect, protection, and fulfillment of human rights contribute to positive sexual health outcomes. This document was the basis to evaluate whether Philippine normative documents such as laws, policies, and guides conform with human rights standards. The WHO document has nine summary recommendations or headers, namely non-discrimination, availability, accessibility, acceptability, quality, informed decision-making, privacy and confidentiality, participation, and accountability. These headers organize and summarize 24 sub-recommendations. The full text of all recommendations and sub-recommendations are in Table 2 in the results section.

### Selection of documents

This assessment was to generate a broad and current list of normative documents to review, using three sets of procedures and approaches. The first and core set of documents included the main national laws and policies on reproductive health, namely, the current RH law (2012), its latest implementing rules and regulations (2017), the various Supreme Court decisions on the law (2014–2017), and a Presidential order for the law’s strict implementation (2017). One of the WHO recommendations included access to safe abortion, and for this, the document used was the Revised Penal Code’s section on abortion (1930). These are the core normative documents on contraception and reproductive rights in the Philippines [11, 12, 14–19]. We also reviewed the DOH’s latest family planning clinical practice guidelines and its postpartum supplement (2014, FP CPG), and the Department of Education’s curriculum guide on health (2016). These documents contain the country’s specific guidelines on contraceptive services, information, and education [20–22].

The second set of documents came from those used in the DOH’s Adolescent and Youth Health Programme [23]. These include a training manual on adolescent health, which contains standards for adolescent-friendly care; an adolescent job aid manual; and guidelines on behaviour change communication strategies for preventing adolescent pregnancies [24–26].

The next set of documents was from the DOH’s online database of policies [27]. A search strategy in the database included keywords “reproductive health,” “family planning,” “contraceptive,” “contraception,” “adolescent,” “youth,” “HIV,” and “AIDS.” We further narrowed the search on policies classified as an “administrative order,” and on those from 1990 up to 2017. We removed documents that were not on contraception, or those that have been superseded by the RH law and its implementing policies.

### Classification based on WHO’s summary recommendations and sub-recommendations

Each document was reviewed in relation to the WHO human rights recommendations and assessed if it targeted adolescents. We then summarized and classified the normative documents’ congruence with the WHO’s sub-recommendations based on the following five categories:

A. - Normative guidance specific to adolescents is present and in agreement with WHO sub-recommendations
B. - Normative guidance for the general population but relevant to adolescents is present and in agreement with WHO sub-recommendations
C. - Normative guidance on WHO sub-recommendations is not present
D. - Normative guidance for the general population but relevant to adolescents is present, but not in agreement with WHO sub-recommendations
E. - Normative guidance specific to adolescents is present, but not in agreement with WHO sub-recommendations

This scale was expanded from that developed by Hoopes et al. in analyzing normative documents in South Africa [28], using three categories. We added the last two categories to reflect the status of Philippine policies more appropriately.

As a final step, we classified normative guidance in the country as in agreement, partly in agreement, or not in agreement with WHO’s summary recommendations. We used “in agreement” when all sub-recommendations are in group A or B; “partly in agreement” when only part of the sub-commendations are in group A or B; and “not in agreement” when all are in group D or E.

### Handling of conflicting provisions

During the review and assessment, we found a few cases wherein topics or legal provisions were in conflict. One example is the FP clinical practice guidelines (CPG) having a section on the use of emergency contraception (EC). However, the RH law prohibits the availability and use of EC in government hospitals. In these few cases, we added enforceability as a factor in categorization. Based on the country’s legal system, documents are broadly classified into three types based on levels of enforceability [29]. From the highest to the lowest level of enforceability, these are laws, implementing policies, and technical guides or guidelines. Laws include the current constitution, decisions of the Supreme Court, and legislative statutes. Implementing policies include executive orders, implementing rules and regulations and administrative orders. These policies should be based on specific laws and are used to run and administer offices and programmes. Technical guides or guidelines include training guides, clinical practice guidelines, operations manuals, school curricula, and best practice recommendations. In the above example, the RH law takes precedence over the FP CPG.

### Analysis team

A table of the key statements from the documents and assessments was developed and circulated to all 5 authors. Three Filipino authors reached a consensus on the category for each sub-recommendation after several rounds of reviews and discussions. Another Filipino expert on Family Planning served as an external reviewer who validated the findings. Other authors helped with the analysis and clarification.

## Results

Based on the search strategies and the three selection procedures described above, we used nine documents selected for review [30–38] with 23 normative guidance documents. Except for the penal code, these were issued from 2009 to 2017. Table 1 lists these documents in full, grouped by selection procedure used and sorted chronologically.

**Table 1:** List of normative documents reviewed for this study grouped by search selection procedure used and chronologically [separate file]

Documents used in the review of laws, policies, and guidance	Date of publication	Type of document
I. National Laws and Policies on Reproductive Health		
Food and Drug Administration (FDA). Advisory 2017–302: Results of the FDA’s Re-evaluation of Contraceptive Products for Recertification (in compliance with the Supreme Court’s April 2017 decision in ALFI et al. vs. Garin et al. and Noche et al. vs. Garin et al.)	Nov 2017	Implementing policy
Revised Implementing Rules and Regulations (IRR) of the Responsible Parenthood and Reproductive Health Act of 2012 (R.A. No. 10354)	Nov 2017	Implementing policy
Supreme Court of the Philippines, Second Division. Decision on Alliance for the Family Foundation, Philippines, Inc. (ALFI) and Atty. Maria Concepcion S. Noche, et al. vs. Hon. Janette L. Garin, et al./Maria Concepcion S. Noche, et al. vs. Hon. Janette L. Garin, et al.	Apr 2017	Supreme court decision and law
Office of the President. Executive Order No. 12, s. 2017: Attaining and Sustaining “Zero Unmet Need For Modern Family Planning” Through the Strict Implementation of the Responsible Parenthood and Reproductive Health Act, Providing Funds Therefor, and for Other Purposes.	Jan 2017	Implementing policy
Supreme Court of the Philippines, Second Division. Decision on Alliance for the Family Foundation, Philippines, Inc. (ALFI), et al. vs. Hon. Janette L. Garin, et al./Maria Concepcion S. Noche, et al. vs. Hon. Janette L. Garin, et al.	Aug 2016	Supreme court decision and law
Department of Education. K to 12 Curriculum Guide: Health (Grade 1 to Grade 10)	2016	Technical guide
Supreme Court of the Philippines (en banc). Decision on Imbong J. et al. vs. Ochoa P. et al.	Apr 2014	Supreme court decision and law
DOH. The Philippine Clinical Standards Manual on Family Planning (2014 Edition)	2014	Technical guide
DOH. Postpartum Family Planning: Supplement to the Philippines Clinical Standards Manual on Family Planning (2014 Edition)	2014	Technical guide
Republic Act No. 10354: An Act Providing for a National Policy on Responsible Parenthood and Reproductive Health	Dec 2012	Law
Revised Penal Code (Act No. 3815): Articles 256–259	1930	Law
II. Materials from the DOH Adolescent and Health Programme		
DOH. Behavior Change Communication Strategies for Preventing Adolescent Pregnancy: Sourcebook	2012	Technical guide
DOH. Adolescent Job Aid Manual: Desk Reference for Primary Level Health Workers in the Philippine Setting (Adapted from the World Health Organization in Collaboration with the Society of Adolescent Medicine in the Philippines, Inc.)	2009	Technical guide
Department of Health (DOH). Competency Training on Adolescent Health for Health Service Workers: A Reference Material	Undated	Technical guide
III. Materials from the DOH online database of policies, focusing on reproductive health and youth		
DOH. AO 2017–0019: Policies and Guidelines in the Conduct of Human Immunodeficiency Virus (HIV) Testing Services (HTS) in Health Facilities	Sep 2017	Implementing policy
DOH. AO 2016–0041: National Policy on the Prevention and Management of Abortion Complications (PMAC)	Nov 2016	Implementing policy
DOH. AO 2016–0005: National Policy on the Minimum Initial Service Package (MISP) for Sexual and Reproductive Health (SRH) in Health Emergencies and Disasters	Feb 2016	Implementing policy
DOH. AO 2015–0002: Creation of a National Implementation Team (NIT) and Regional Implementation Teams (RIT) for Republic Act 10354 (Responsible Parenthood and Reproductive Health Law of 2012)	Jan 2015	Implementing policy
DOH. AO 2014–0046: Defining the Service Delivery Networks (SDNs) for Universal Health Care or *Kalusugan Pangkalahatan*.	Dec 2014	Implementing policy
DOH. AO 2014–0042: Guidelines on Implementation of Mobile Outreach Services for Family Planning	Oct 2014	Implementing policy
DOH. Administrative Order (AO) 2013–0013: National Policy and Strategic Framework on Adolescent Health and Development	Mar 2013	Implementing policy
DOH. AO 2012–0009: National Strategy Towards Reducing Unmet Need for Modern Family Planning as a Means to Achieving MDGs on Maternal Health	Jun 2012	Implementing policy
DOH. AO 2011–0005: Guidelines on Ensuring Quality Standards in the Delivery of Family Planning Program and Services Through Compliance to Informed Choice and Voluntarism	Jun 2011	Implementing policy

Of nine WHO summary recommendations, Philippine normative documents are in agreement with four, namely on acceptability, participation, accountability, and quality. Documents with the first three recommendations had provisions specific to adolescents (Category A), with those with the last one had provisions for the general population, but relevant to adolescents (Category B). Philippine normative documents are partly in agreement with the remaining five WHO summary recommendations, namely on nondiscrimination, availability, accessibility, informed decision-making, and privacy.

Of twenty-four WHO sub-recommendations, Philippine normative documents are in agreement with fifteen, are partly in agreement with four, and are not in agreement with five. Table 2 displays the full analyses of the various policy documents in relation to the WHO standards, which will be described in more detail below.

**Table 2:**
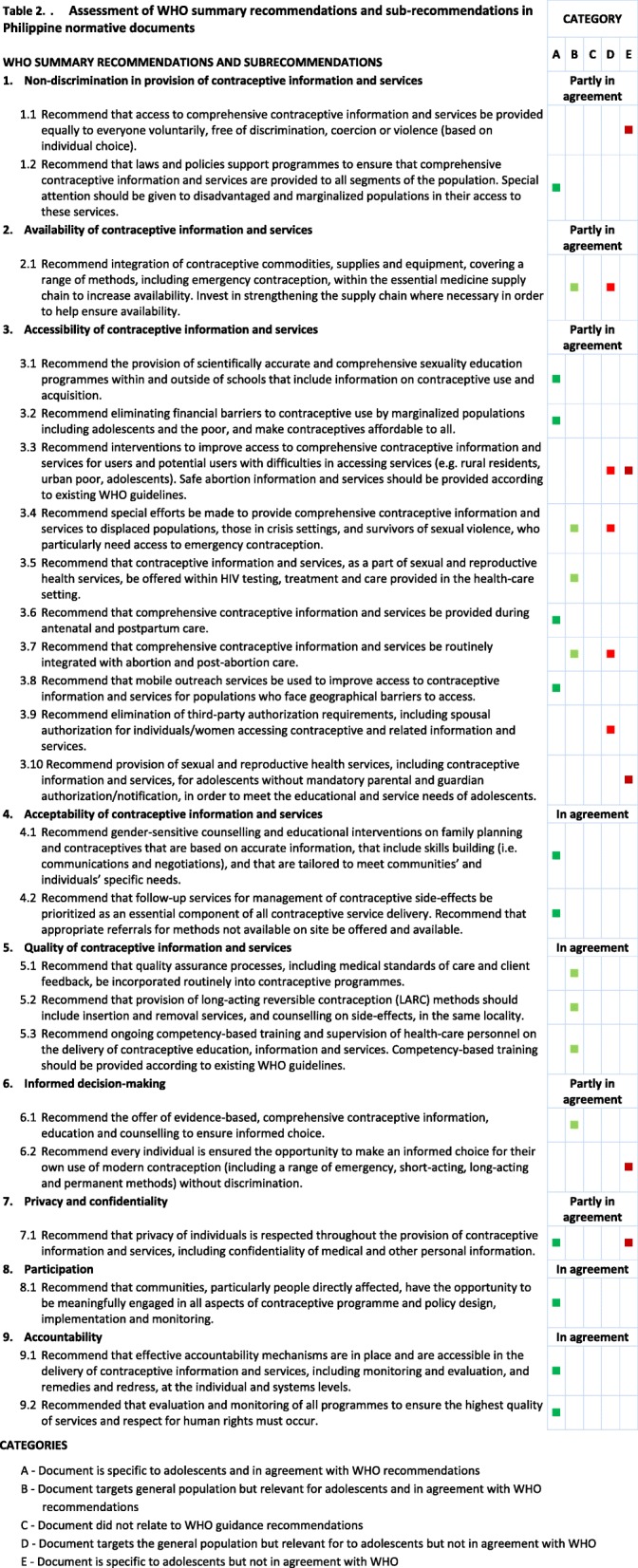
Assessment of WHO summary recommendations and sub-recommendations in Philippine normative documents

### Non-discrimination: Partly in agreement with summary recommendation

#### Non-discrimination in information and services (e)

The RH law invokes respect for human rights of all persons and non-discrimination explicitly and repeatedly. However, it does not allow minors access to modern contraception without “written consent from their parents or guardian/s” [11]. The law’s restriction overrides a Presidential order to accelerate family planning (FP) and to achieve “zero unmet need for modern contraception” [17].

#### Special attention to disadvantaged and marginalized populations (a)

The RH law calls for prioritizing the needs of women, children, and other underprivileged and vulnerable sectors [11]. An implementation policy for adolescent health and development mandates equity and inclusion for marginalized and vulnerable adolescents [36].

### Availability: Partly in agreement with summary recommendation

#### Integration of contraceptives, including emergency contraception, into essential medicines (b, d)

The RH law mandates that modern contraceptives should be certified as essential medicines, and should be purchased and distributed by the government throughout the country. [11]. The Food and Drug Administration (FDA) must first certify that the contraceptive is not an abortifacient, defined as “any drug or device that induces abortion, or the destruction of a fetus inside the mother’s womb, or the prevention of the fertilized ovum to reach and be implanted in the mother’s womb.” Budgets and logistics are specified per level of the service delivery network at all levels, from outposts and primary health centers to hospitals [15, 34]. However, government hospitals and local government health facilities are not allowed to purchase or acquire emergency contraceptive pills (ECP) [11]. Private facilities are not expressly included in the prohibition; therefore, the FP CPG on ECPs would apply [20].

### Accessibility: Partly in agreement with summary recommendation

#### Scientifically accurate, comprehensive sexuality education (a)

The RH law requires “age- and development-appropriate” RH and life skills education for adolescents in formal and non-formal schools [11]. Policies mandate the inclusion of “gender-sensitive and rights-based” sexuality education in the curriculum [15], and modern FP methods in the education department’s K to 12 standards [22].

#### Elimination of financial barriers to contraceptive use by marginalized populations (a)

The RH law requires the health department to provide free contraceptive supplies to poor and marginalized families [11]. An implementation policy asks for the development of an “adolescent health benefits package” in the social health insurance system. The same policy calls for the mobilization of local government and private funds to ensure the provision of health services and FP supplies for adolescents [36]. A technical guide on adolescent-friendly health care services recommends free health services for adolescents [26].

#### Improving access for those with difficulties in accessing services, including safe abortion according to existing WHO guidelines (e, d)

The RH law provides various mechanisms to overcome geographic, financial and social barriers and thereby facilitate access to contraceptive information and services [11, 15]. However, contraceptive access by adolescents is partly constrained by the requirement for parental consent and the restriction on ECP procurement and distribution. Induced abortion is prohibited by the general penal code, with no explicit exception allowing conditional use [19].

#### Special efforts for displaced populations, in crisis settings and survivors of sexual violence (b, d)

The RH law mandates access to contraceptive information and services by people in difficult circumstances, including survivors of violence and those in crisis and disaster situations. A policy describing a Minimum Initial Service Package (MISP) directs the government to provide these and other reproductive health services during disasters [11, 32]. The FP CPG recommends the provision of regular and emergency contraceptive services during emergency and crisis situations [20]. However, legal restrictions to minors and the use and availability of emergency contraception are still in place [11].

#### Contraceptive information and services within HIV testing, treatment and care (b)

The RH law defines a package of 12 RH care elements that include FP, adolescent RH (ARH), and the prevention and management of STIs, HIV, and AIDS [11]. Integrated services at all levels of the health care delivery system are directed by law’s implementing policies [15, 34]. The FP CPG includes integration guidance for FP providers [15].

#### Contraceptive information and services during antenatal and postpartum care (a)

The RH law and its implementing rules define and mandate an integrated package that includes contraception, ARH, antenatal and postpartum care which must be provided in a service delivery network [11, 15, 34]. The FP CPG and its postpartum FP supplement provide guidance on the provision of postpartum contraception [20, 21]. The technical guidance on adolescent health services discusses counseling on contraception for pregnant adolescents [25].

#### Contraceptive information and services routinely integrated with abortion (d) and post-abortion care (a)

The RH law reiterates the penal code’s prohibition on abortion. However, it mandates the treatment of post-abortion complications in a “humane, non-judgmental and compassionate manner” as part of the RH care package [11]. The health department’s post-abortion policy orders the provision of supportive counseling and full access to contraception [31]. The FP CPG recommends a range of contraceptive options post-abortion [20].

#### Mobile outreach services to improve access to contraceptive information and services (a)

The RH law recommends the deployment of Mobile Health Care Service vehicles to deliver contraceptive supplies and services to hard-to-reach and underserved areas [11]. Implementing policies define the mechanisms for operating and sustaining these vehicles [15]. For adolescents, a technical guide recommends outreach services for those in hard-to-reach areas [26].

#### Elimination of spousal authorization (d)

The Supreme Court’s decision on the RH law requires spousal consent, although there are no penalties for health providers who skip this procedure [11, 12]. The FP CPG which are issued after the Supreme Court ruling, requires “spousal consent… prior to undergoing permanent surgical contraceptive methods” [20].

#### Elimination of parental and guardian authorization (e)

The Supreme Court decision requires all minors to have parental consent to access contraceptive services in public facilities, although there are no penalties for skipping it [11, 12]. The FP CPG reiterates this requirement [20].

### Acceptability: In agreement with summary recommendation

#### Gender-sensitive counselling, education, based on accurate information, with skills building tailored to needs (a)

An implementing policy mandates gender-sensitivity training for health providers to develop respect for privacy and confidentiality, and non-judgmental attitudes. Training must also include building sensitivity to the particular needs of adolescents, building counseling skills, and mechanisms of referral of victims of gender-based violence. Information for all patients must be “scientific, correct, evidence-based and comprehensible” [15].

#### Management of side-effects; appropriate referrals (a)

The FP CPG describes the standard management of contraceptive side effects, including those specific to adolescents [20]. The RH law’s implementing policy requires health providers to ensure that referred patients are seen by another health provider “within the same hour” [15]. The Supreme Court allowed “conscientious objectors” to refuse to refer, except when the patient is in an emergency [12]. However, the implementing policy contains detailed steps that a provider must follow—including registration and public notice—before acquiring a conscientious objector status [15].

### Quality of contraceptive information and services: In agreement with summary recommendation

#### Quality assurance processes, including medical standards of care and client feedback (b)

The RH law requires quality of care in service provision [11]. The law’s implementing policy include client-side factors in monitoring and evaluating services, such as cultural preferences, time and financial limitations, distance from facilities, and perception on the conduct of health providers [15, 34]. The FP CPG emphasizes informed choice and respect for clients’ rights in its quality assurance guidance [20].

#### Quality in long-acting reversible contraceptives or LARCs (b)

The FP CPG instructs providers that counseling should explain the benefits and side effects of LARCS, as well as the procedures for their insertion and removal. It also clarifies that requests for removal should not be refused or delayed [20].

#### Competency-based training and supervision of health care personnel (b)

The RH law’s implementing policy requires the health department to conduct baseline competency assessments, competency-based trainings, and regular monitoring and evaluation of all providers to ensure quality of care [15]. The FP CPG discusses supportive supervision, post-training evaluation and monitoring, and regular updates for healthcare providers [20].

### Informed decision-making: Partly in agreement with summary recommendation

#### Evidenced-based information, education, and counseling to ensure informed choice (b)

The RH law emphasizes informed choice as a guiding principle, which is defined and elaborated by a specific policy on informed choice and voluntarism [11, 38]. The law’s implementing policy requires the health department to develop local language information materials on contraception, including contraindications and side effects. These must be scientifically correct, evidence-based and comprehensible [15].

#### Making informed choices without discrimination (e)

The RH law’s implementing policy requires all public health facilities to provide full contraceptive information and services that are “age-, capacity-, and development- appropriate.” These must be available to all clients regardless of “age, sex, gender, disability, marital status, or background” [15]. An implementing policy emphasizes the human rights of adolescents “to have control over and decide freely and responsibly on matters related to their sexuality, including sexual and reproductive health.” The rights of the adolescents include access to “life-saving interventions, as long as he/she is mature enough to face the consequences” [36]. However, the requirement for written parental consent before minors can access contraceptive services severely restricts informed choice [11, 12].

### Privacy and confidentiality: Partly in agreement with summary recommendation

#### Privacy and confidentiality (a, e)

One of the technical guides contains standards on respecting the right to privacy of adolescents [26]. However, the standards do not include contraceptive services. Another technical guide advises that when there is a conflict between restrictive policies and the adolescent’s best interests, the provider needs to draw on his or her personal experience and other knowledgeable people [25]. However, the law’s requirement for parental consent limits the privacy and confidentiality rights of minors who want contraceptives [11, 12].

### Participation: In agreement with summary recommendation

#### People’s engagement in policy design, implementation, monitoring (a)

The RH law mandates the active participation of young people’s organizations in sexual and reproductive health programmes [11]. Two implementing policies mandate self-empowering activities and participation in governance as vital means for achieving SRH [15, 33]. A Presidential order tasks its youth arm, the National Youth Commission, to integrate adolescent reproductive health with youth development programmes [17].

### Accountability: In agreement with summary recommendation

#### Effective accountability mechanisms in place and accessible (a)

The RH law upholds choice and human rights. It prohibits and penalizes acts that prevent access to RH information and services, or those that induce or coerce anyone to use such services. The law enumerates government officials, health providers, employers and private companies as potential violators [11, 12]. The law’s implementing policy requires an RH Officer in all facilities who must receive and act on complaints regarding violations of the law. The implementing policy also tasks the Commission on Human Rights to receive complaints [15].

#### Evaluation and monitoring to ensure quality and human rights (a)

The RH law requires the health department to submit progress reports every year, and participate in an oversight review by the legislature every five years. The law’s implementing policy defines monitoring standards for the RH programme within a service delivery network [15, 34]. A Presidential order elaborates further the monitoring and reporting of FP services [17]. For adolescents, a technical guide recommends the creation of a national technical working group to monitor and evaluate compliance with set standards [26].

## Discussion

Certain parts of the Philippines normative documents are strongly in agreement with the human rights of adolescents in contraception. These include the focus on disadvantaged and vulnerable groups and persons; attention to critical health system elements such as staffing, financing, and supply chains; the integration of RH services in all levels of the health system; and the explicit call to citizens’ participation and accountability.

However, certain parts of existing laws and policies impose substantial restrictions on human rights. These include the requirement for parental and spousal consent; the prohibition on emergency contraceptives in public hospitals; the need for certification that contraceptives are not abortifacients; and the wide latitude given to conscientious objectors. The penal code criminalizes abortion with no explicit exception—none for rape, health risks or life-threatening pregnancies.

### The RH law and conservative political movements

The restrictive parts of Philippine norms are rooted in conservative beliefs and values espoused mainly by the Catholic hierarchy and the so-called “pro-life” movement. Conservatives believe that modern contraception thwarts the natural procreative process, destroys embryonic life, undermines the family, weakens parental rights over children, and promotes sexual license. These religious beliefs are used in political action by advocates adept at working and influencing the executive, legislative and judicial departments [39, 40]. In 1987, they successfully introduced a policy in the Philippine constitution that protects “unborn” life. It commits the State to “equally protect the life of the mother and the life of the unborn from conception.”

Since then, this provision has been used to block or roll back progressive initiatives in SRH [12, 41, 42]. Pro-life chief executives banned FP services in the City of Manila and other local governments (2000–2010). The FDA delisted an emergency contraceptive pill and the multi-use drug misoprostol after petitions by pro-life groups (2001–2002). The health department focused on “natural” FP methods (2003–2010) when a politician close to Catholic bishops became President. The education department stopped a pilot module on adolescent sexuality education after pro-life groups tied it up in court litigation (2009).

The passage of the RH law in 2012 signals a shift in public opinion. A comfortable majority of the population now accepts publicly-funded SRH programmes, including FP. However, challenges to the law persist, mainly through the courts. In 2015, a pro-life group convinced the Supreme Court to stop the government’s use of progestin implants and to recall the non-abortifacient certifications issued to 48 contraceptives [16, 18]. The restraining order lasted over two years, caused shortages in contraceptive supplies and distribution and denied some women their preferred method [40]. The FDA restarted their process and ruled again in November 2017 that all questioned contraceptives were non-abortifacient [14], which lifted the restraining order. Soon after, a network calling itself Pro-Life Coalition began an online signature campaign to reverse the FDA decision.

### Controversy about sexuality education and contraception education for adolescents

The RH law envisions the inclusion of adolescents in the RH programme, but mainly through education and counseling. While the law mentions RH services, it is silent on contraception for adolescents. There is a strong mandate to provide comprehensive RH education in all mainstream and alternative schools. The curriculum must be age- and development- appropriate. The comprehensive and developmental approach would correct the old practice of doing isolated lessons in specific grades, such as teaching contraceptive methods in Grade 10. The law identifies critical subjects that should be taught but does not explicitly include sexuality and contraception. It advises flexibility in deciding topics and methodology based on consultations with stakeholders like parents and other “interest” groups.

Two reasons can explain this caution. In 2009, the education department was stopped and brought to court by a pro-life group for pilot-testing a high school sexuality education module. Though the education department eventually won the case, it never reintroduced the module [40]. In 2014, pro-life groups argued that sexuality education violates the primary duty of parents over their children, which makes the RH law unconstitutional. The Supreme Court dismissed this argument for being premature as there was no curriculum yet to oppose [12]. Pro-life groups could revive their case once a comprehensive curriculum is released.

The technical guides on contraceptive services and information reflect the equivocation of the law. Most guidance materials recommend abstinence as the best behavior for all adolescents regardless of their specific life situation. The guides are silent on relevant factors such as age, marital status, experience of sexual violence, and capacity for responsible decision-making. They promote abstinence-only or abstinence-centered values and practices. A representative guide, the Behavior Change Communication sourcebook [24], presents abstinence as the best way to prevent adolescent pregnancies; advises sexually active adolescents to return to abstinence; and recommends the exclusion of abstinent adolescents from public education sessions on contraception for fear they get the misimpression that sex is permissible as long as it is protected.

#### Devolution and health policies system

The Philippine health system is highly decentralized with significant powers and functions transferred to 1600 local governments (i.e., provinces, cities, and municipalities). The restructuring was part of a broader government devolution mandated by the Constitution and implemented by a 1991 law [43, 44]. Devolved health functions include financing and budgeting, operating facilities from health posts to provincial hospitals, hiring and managing health personnel, and creating local health policies and programmes. There are, in effect, two parallel health systems: national and local health systems.

The national health department develops policies for the national health system and operates some tertiary care hospitals. Local health offices implement programmes under the authority of local chief executives. Without a national law, the concept of “local autonomy” provides local chief executives the power to ignore or sideline national health policies and programmes. For example, the mayor of Manila banned contraceptive services in local health facilities in 2000 because of his objections based on his religious beliefs. It took a new mayor to partly restore services in 2012 [45]. Local officials may also refuse to cooperate with other local officials because of political or personal differences.

This situation can result in a disparate, poorly-integrated health system that could also account for the country’s stagnating performance in areas like tuberculosis control, immunization, FP and maternal mortality reduction. The health department has identified fragmentation as a critical problem for many years now but has been unable to address it because of local government autonomy. The RH law and its implementing policies provide measures for health system strengthening and integration, but only an amendment of the devolution law may provide a strategic solution.

## Conclusion and recommendations

Many Philippine norms are in agreement with adolescents’ human rights to contraceptive information and services as recommended by the WHO. However, a significant number are restrictive, reflecting the strong influence of conservative religious beliefs.

To continue progress, we recommend the following:

1. Encourage the further elaboration of policies that are in agreement with WHO recommendations, and based on scientific evidence

- Encourage government offices involved in implementing the RH law to create policies for institutionalizing the involvement of adolescents and young people decision-making on SRH, particularly contraception, and provide for their training and ongoing support.
- Support the education department’s efforts to shift from an abstinence-only framework to comprehensive sexuality education in its K to 12 curriculum through targeted technical support.
- Update the health department’s policies to enable the provision of contraceptive services to adolescents which are allowed by the RH law, such as services to adolescents aged 18 and above; minors who have consent from their parents or guardians; and minors consulting in private and NGO facilities, which are not explicitly covered by the prohibition in the law.
- Flesh out the health department’s guidelines on the financing of adolescent health services with a policy specifying how different agencies, notably the social health insurance agency, national health units, local governments, and other funding partners can support contraceptive services to adolescents who are legally qualified to receive such services.
- Amend the health department’s technical guide on behavior change communications to remove its abstinence-centered and sex-negative content and to be in agreement with the education department’s Comprehensive Sexuality Education framework.

2. Encourage the legal clarification of restrictive parts of the RH law based on Section 27 which states that the law must be “liberally construed to ensure the provision, delivery and access to reproductive health care services, and to promote, protect and fulfill women’s reproductive health and rights.”

- Issuance by the health department of a legal opinion clarifying that while the law recognizes spousal consent, it does not include penalties for those who prefer to omit this procedure.
- Issuance by the health department of a legal opinion clarifying that the prohibition on the procurement, distribution, and provision of emergency contraception pertains only to government hospitals and therefore does not apply to private and nongovernment providers.

3. Explore the amendment of restrictive laws or restrictive parts in these laws.

- Conduct legal research, including on the impact of restrictive policies and legal options.
- Popularize ethical and human rights norms.

### Limitations

The research focused on the content of normative documents, not their actual implementation in practice. It is possible that reality may not correspond with what is written. The study occurred amidst two major political changes: the national elections in mid-2016 that resulted in a new set of elected officials at the highest levels; and Supreme Court rulings on contraception in 2015, 2016, and 2017, which affected contraceptive access generally. We incorporated new documents released during these years but may have missed important developing changes or trends.
